# EMLA-Induced Skin Wrinkling for the Detection of Diabetic Neuropathy

**DOI:** 10.3389/fneur.2013.00126

**Published:** 2013-09-02

**Authors:** Kay Wei Ping Ng, Jonathan J. Y. Ong, Thaw Dar Nyein Nyein, Shen Liang, Yee Cheun Chan, Kok Onn Lee, Einar Patrick Wilder-Smith

**Affiliations:** ^1^Division of Neurology, Department of Medicine, National University Hospital, Singapore; ^2^Biostatistics Unit, Yong Loo Lin School of Medicine, National University of Singapore, Singapore; ^3^Division of Endocrinology, Department of Medicine, National University Hospital, Singapore

**Keywords:** skin wrinkling, diabetic polyneuropathy, nerve conduction study, water-immersion wrinkling, EMLA

## Abstract

**Objective:** To determine the usefulness of Eutectic Mixture of Local Anesthetic (EMLA)-induced stimulated skin wrinkling (SSW) to detect diabetic sensorimotor polyneuropathy (DSPN).

**Research Design and Methods:** Two hundred and ten diabetics were prospectively recruited (mean age 58.5 ± 12.7 years) from a large tertiary center from 2009 to 2011. EMLA was applied to the tips of digits 2, 3, and 4 and the degree of wrinkling graded. Diabetic Neuropathy Symptom (DNS) score, nerve conduction studies (NCS), Semmes–Weinstein monofilament (SWMF) tests, and vibratory perception thresholds (VPTs) testing were chosen as comparative clinical standards to diagnose length-dependent DSPN.

**Results:** Inter-rater agreement for two examiners of SSW was high, with Cohen’s weighted *κ* of 0.912 for the right hand, and 0.823 for the left. *K* measure of agreement of SSW with the NCS, DNS scores, SWMF testing, and VPT testing was 0.486, 0.243, 0.289, and 0.395 respectively. SSW was able to distinguish between normal and abnormal NCS and DNS results, with median scores of 3.333 vs. 1.667 (*p* < 0.0005); and 3.167 vs. 2.000 (*p* < 0.0005) respectively. Following receiver operating characteristic-analysis, at a cut-off point of <3 for an abnormal SSW test, sensitivity of SSW test for diagnosing DSPN using NCS as a reference standard was 81.3%, and specificity was 67.0%, on par with other testing methods.

**Conclusion:** SSW shows comparable sensitivity to other methods for detecting DSPN. Given its low cost and easy administration, SSW can be considered a useful alternative screening method for diagnosing diabetic neuropathy.

## Introduction

Stimulated skin wrinkling (SSW), refers to the reversible undulations of the surface skin occurring 5–30 min following water immersion or exposure to a eutectic mixture of local anesthetic (EMLA) (1, 2). Skin wrinkling occurs as a result of vasoconstriction in the glabrous skin, mediated by post-ganglionic sympathetic fibers (2). As a test, it can be a simple method of determining small nerve fiber function (1, 3), and has been shown to correlate with intraepidermal nerve fiber density (IENFD) in patients with a sensory neuropathy (4, 5).

Of the neuropathies developing in diabetic patients, the most common form is a chronic, symmetrical, length-dependent diabetic sensorimotor polyneuropathy (DSPN) (6). There is overall agreement that small nerve fiber involvement occurs earlier than large fiber neuropathy, is common and may even be subclinical (7). Screening for early diabetic polyneuropathy is essential for timely foot care to prevent foot ulcers. At present, abnormal nerve conduction studies (NCS) is advocated as a minimal criteria for the diagnosis of DSPN (8). However, NCS generally requires the consultation of a trained neurologist and/or technologist, and the use of specialized electrodiagnostic equipment, which are often not readily available especially in a general practice setting. NCS also only assesses larger nerve fiber function. Alternative screening methods for diabetic distal polyneuropathy have been developed and used, such as clinical scoring systems (9, 10), including the Diabetic Neuropathy Symptom (DNS) score (11), the Semmes–Weinstein monofilament (SWMF), vibration threshold testing (12), and autonomic tests (13). However, many of these methods have not been well-validated, and may be time-consuming, or require their own specialized equipment.

We sought to determine if SSW with EMLA could be used as a simple diagnostic tool for diabetic neuropathy, especially since small nerve fibers are affected early on in the course of diabetic polyneuropathy (14).

## Research Design and Methods

Diabetic patients, aged 21–80 years were prospectively and consecutively recruited from specialist diabetic and neurology clinics in a tertiary center (National University Hospital, Singapore) over a 2-year period from 2009 to 2011. Patients were recruited based on a confirmed diagnosis of diabetes mellitus and the persons recruiting were not aware of the presence or absence of diabetic polyneuropathy. The institutional review board of the hospital approved the study.

### Patients

After giving informed consent, 210 diabetic patients were recruited. Patients had to fulfill criteria for the diagnosis of Diabetes Mellitus (15). Patients subsequently underwent a detailed medical and neurological evaluation to exclude other discernible causes of large- or small-fiber neuropathy. Exclusion criteria were a history or laboratory evidence of uremia, cancer, past or current chemotherapy, alcohol abuse, solvent or toxic exposure, dementia, spinal cord and root disease, thyroid dysfunction, vitamin B12 deficiency, and presence of peripheral vascular disease or abnormal vascular responses such as Raynaud’s phenomenon. Patients with open wounds over the finger-tips where the EMLA cream would be applied were also excluded.

### Tests of nerve function

Nerve conduction studies, the DNS score, SWMF test, and Vibratory Perception Threshold (VPT) testing using Biothesiometry were chosen as our comparative clinical standards for SSW. All tests were performed using standardized protocols on the same day for each patient. The DNS score was obtained first, followed by the SWMF and VPT tests, then the NCS. The SSW was performed in between these tests depending on the availability of the testing room. Two technicians with more than 5 years of experience and board certification performed all the tests blinded to the clinical status of the patient.

### DNS score

The score has the following items: (i) unsteadiness in walking, (ii) pain, burning or aching at legs, or feet, (iii) prickling sensations in legs or feet, and (iv) numbness in legs or feet. It is scored “1” if a symptom occurred several times a week during the last 2 weeks, and scored as absent “0” if it did not, with the maximum score being 4 points. As per guidelines, diabetic polyneuropathy was scored present with 1–4 points, and absent with 0 points (11).

### Semmes–Weinstein monofilaments

These were tested using the five-piece hand kit on the plantar aspect of the hallux, and the base of the first, third, and fifth metatarsals (with removal of callus when necessary), using standard guidelines (16, 17). Monofilament testing was also conducted on the palmar aspect of the index finger, middle finger, and ring finger. The patient was asked to say “yes” when he or she sensed the application of a monofilament. Three trials were administered for filaments 2.83 (0.07 g) and 3.61 (0.4 g). When the patient was unable to respond correctly in at least one trial, a heavier monofilament (4.31, 4.56, 6.65) was trialed only once (2, 4, and 300 g monofilaments respectively). As per manufacturer, insensitivity to 0.4 g monofilament and lighter was taken as abnormal plantar thresholds. Peripheral polyneuropathy was diagnosed when plantar thresholds of both feet were abnormal.

### Vibration perception threshold testing

Vibratory perception threshold was tested using a hand-held Biothesiometer (Biomedical Instruments, Newbury, OH, USA) performed according to standardized protocols (18, 19). The device was held with the rubber tactor placed perpendicular to the pulp of the terminal phalanx of the thumb, over the pulp of the big toe, and over the medial malleoli. The voltage of vibration was gradually increased from 0 V until the patient could perceive the vibration. This was performed three times, and the mean of the three trials taken to determine the VPT. Age-adjusted reference values were used, with abnormal values considered as at least 2 SDs higher than the mean (19, 20). For a VPT test to be considered indicative of diabetic polyneuropathy, patients had to have abnormal readings over both big toes.

### Nerve conduction studies

Nerve conduction studies testing studied the median, ulnar, peroneal, tibial, and sural nerves of all four limbs. Skin temperature was recorded, and NCS recordings made using a two-channel electromyography machine (Medelec Synergy, Oxford Instruments, Oxford, UK), following suggested standardized nerve conduction study protocols, according to the standards of the American Association for Neuromuscular and Electrodiagnostic Medicine (21).

Parameters evaluated included the distal motor latency (dML), compound muscle action potential (CMAP), amplitude measured from the baseline to the negative peak, mean F-wave latency, distal sensory latency (dSL) measured to the negative peak, and sensory nerve action potential (SNAP) amplitude measured from negative to positive peak. Using standardized distances between electrode placements, conduction velocity (CV) was also calculated. Antidromic testing was carried out for sural nerve testing, while orthodromic testing was performed for median sensory and ulnar sensory testing. An age-standardized normal range determined in our local population was used. Patients were diagnosed with diabetic polyneuropathy when an abnormal sural nerve (dSL and/or SNAP) response was obtained, with an abnormal peroneal (CMAP and/or F-wave) response. Both sural nerves had to be abnormal to meet the case definition of diabetic polyneuropathy (22).

In addition, patients were also evaluated for presence of median neuropathy at the wrist, with additional testing of a second lumbrical-interossei (2L-INT) latency difference. Neurophysiological results were taken as supportive of median neuropathy at the wrist in the case of two of the three following abnormalities: median nerve motor distal latency >4.4 ms, median nerve sensory distal latency >3.4 ms or absent, and a 2L-INT latency difference >0.6 ms (based on our own laboratory norms).

### Stimulated skin wrinkling test

**Figure 1:**
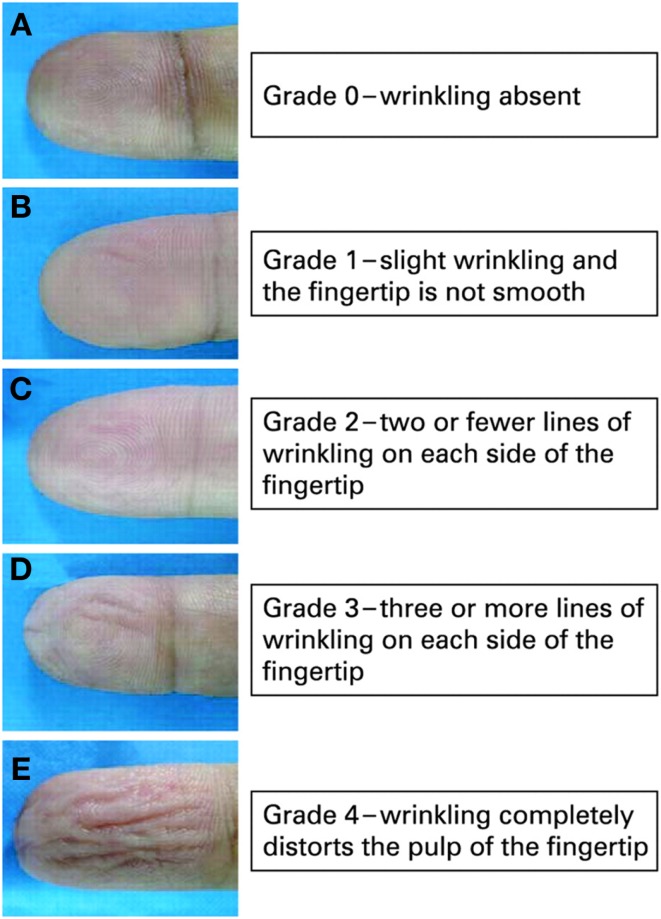
**Wrinkling scale**.

One milliliter of EMLA (lidocaine 2.5% and prilocaine 2.5%, AstraZeneca), or the amount needed to thickly and completely cover the distal digit pulp, was applied evenly onto the finger-tips of the index finger, middle finger, and ring finger (digit 2, 3, and 4) of both hands. This was covered with Tegaderm^®^ tape for 30 min. Photographic documentation pre-EMLA and post-EMLA was performed. Wrinkling was graded using a previously published scale and was based on the assessment of the photographic picture obtained (Figure 1) (4). Previously, we had established that skin wrinkling in response to EMLA in normal subjects was grade 3 and 4 (1) (skin wrinkling was therefore considered abnormal if absent or severely impaired; Grades 0–2). The grading of wrinkles were: Grade 0, wrinkling absent; Grade 1, just perceptible wrinkling, with the fingertip not completely smooth; Grade 2, two or less lines of superficial wrinkling on the fingertip; Grade 3, three or more lines of deep wrinkling on the fingertip; Grade 4, wrinkling completely distorts the pulp of the fingertip. Wrinkling grades for digits 2, 3, and 4 were totaled and averaged. A difference of ≥3 marks per hand (i.e., ≥1 mark per digit) was taken as cut-off for a different score. A score of ≥9 marks for each hand was taken as normal and without evidence of neuropathy. Patients were not allowed to apply any hand cream in the 1-h preceding testing. SSW evaluation was performed blinded as the assessment was performed without the knowledge of the other results.

In order to assess test reproducibility, two trained examiners blinded to the patient’s clinical status, graded the skin wrinkling scores in 46 of these patients, both on the same day of testing, under identical conditions.

The following data variables was also collected: current smoking history, presence of hypertension, recent ingestion of coffee or tea within the preceding 3 h of the SSW test, and skin temperature.

### Statistical analysis

All statistical analyses were performed using SPSS-PC statistical software (version 20.0). Student’s *t*-tests were used to compare continuous data between patients with normal and abnormal SSW tests, while Fisher’s exact test performed for categorical data. *p* Values <0.05 were considered significant. Receiver Operating Characteristic (ROC) curve analysis was used to determine the operating characteristics of the various clinical methods tested on the identification of patients with diabetic polyneuropathy, using NCS criteria as the reference standard. Inter-rater agreement was assessed using Cohen’s weighted κ. Unless stated, hands and feet were analyzed as a pair.

## Results

Two hundred and ten diabetic patients were recruited. Fourteen patients had missing data for DNS scores, one had missing data for SSW, one had missing data for NCS, and two had missing data for VPT. Patient characteristics are summarized in Table 1.

**Table 1:** **Patient characteristics**.

Age (years)	58.5 ± 12.7 (21–80)
Males	41.4
Duration of DM (years)	7.33 ± 6.54 (1–33)
Diabetes type (Type 1/Type 2)	8.22/91.78
HbA1c	8.36 ± 1.93 (5.6–13.6)
Presence of retinopathy	12.0
Presence of nephropathy	40.0
Abnormal NCS consistent with DSPN	53.6
Abnormal DNS scores	67.3
Abnormal SWMF tests consistent with DSPN	28.1
Abnormal VPT testing consistent with DSPN	72.1
Abnormal SSW scores	58.9

*Data are in percentage, or means ± SD (ranges)*.

### Reproducibility

We have previously demonstrated a high inter- and intra-rater correlation for the SSW assessment with EMLA (5). In this current study, 2 trained examiners blinded to the patient’s clinical status, graded the skin wrinkling scores in 46 of these patients, both on the same day of testing, under identical conditions. Inter-rater agreement showed a Cohen’s weighted κ for both raters of 0.912 for the right hand, and 0.823 for the left hand, indicating a good level of agreement.

### Relationship of SSW with the tests of nerve function

*K* measure of agreement between SSW test and the studied tests of nerve function was calculated. SSW test had best agreement with NCS compared to the other screening methods. Agreement scores with the NCS, DNS scores, SWMF testing, and VPT threshold testing were 0.486, 0.243, 0.289, and 0.395 respectively.

### Relationship of SSW with NCS and DNS as reference standards

**Figure 2:**
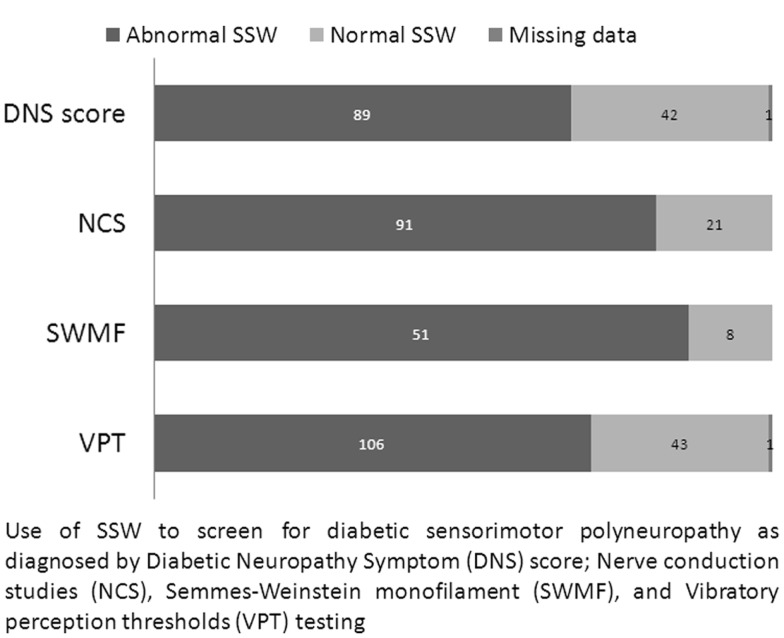
**Comparison of stimulated skin wrinkling with other test parameters**.

Stimulated skin wrinkling was able to predict the presence of abnormal NCS or DNS results, with a median SSW score of 3.333 vs. 1.667 for normal (*n* = 97) and abnormal NCS (*n* = 112) respectively (*p* < 0.0005); and a median SSW score of 3.167 vs. 2.000 for normal (*n* = 64) and abnormal DNS score (*n* = 132) respectively (*p* < 0.0005). Figure 2 shows the relationship of SSW with other nerve tests.

### Sensitivity and specificity using NCS as the reference standard

**Figure 3:**
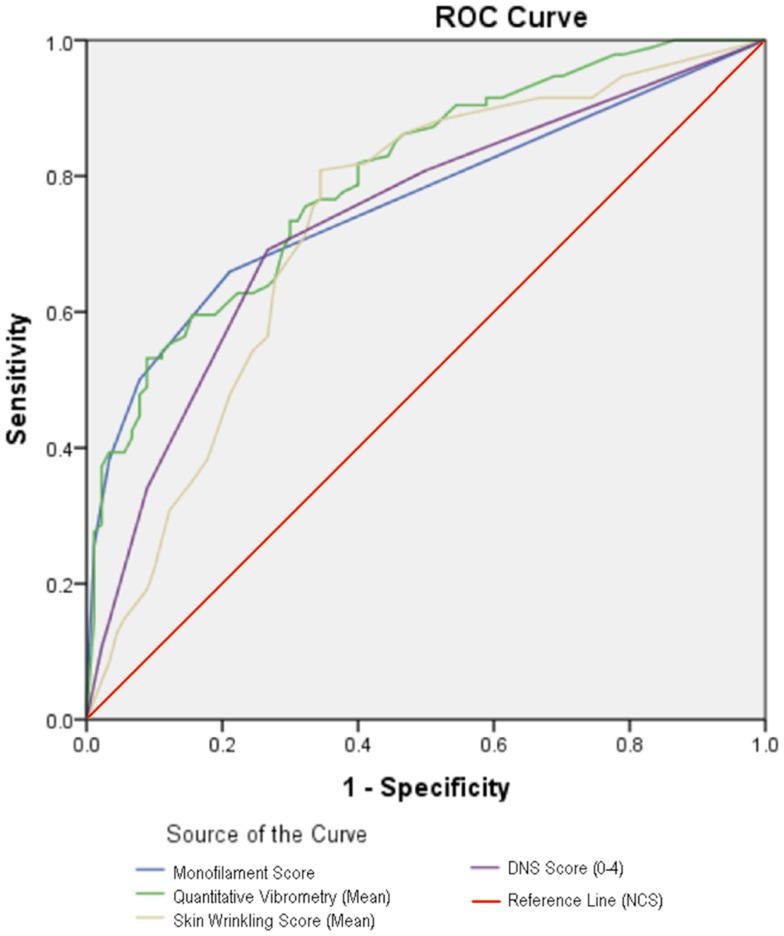
**ROC of various test parameters**.

Figure 3 shows the ROC curves of the clinical tests of nerve function, when compared with NCS. The areas under the curve for SWMF testing, VPT testing, and DNS scores were 0.760, 0.798, and 0.735 respectively. Area under the curve for SSW test was 0.732, comparable to that of DNS scoring.

At a cut-off point of <3 as an abnormal SSW test, using NCS as the reference standard, sensitivity of SSW test for diagnosing DSPN was 81.3%, and specificity was 67.0%. This put SSW on par with VPT testing, with the highest sensitivity and specificity of the clinical tests studied.

### Variables associated with stimulated skin wrinkling test

Of the variables studied, an abnormal SSW test was associated with older ages and lower skin temperatures (Table 2).

**Table 2:** **Variables associated with SSW testing**.

	Normal SSW test	Abnormal SSW test	*p* Value
Mean age (years)	54.80 ± 13.77	60.26 ± 11.31	0.003
Mean temperature (°C)	31.94 ± 1.70	31.34 ± 2.01	0.025
Males	47 (39.8)	71 (60.2)	0.673
Current smoking	10 (38.5)	16 (61.5)	0.833
Hypertension	56 (40.3)	83 (59.7)	0.767
Coffee/tea intake in preceding 3 h	14 (60.9)	9 (39.1)	0.071
Presence of median neuropathy at the right wrist	26 (32.5)	43 (37.4)	0.544
Presence of median neuropathy at the left wrist	27 (31.8)	35 (31.2)	1.000

*Data are means ± SD or *n* (%)*.

## Discussion

This large study of diabetic patients is the first to demonstrate that SSW is a valid and reliable tool for detecting the presence of diabetic neuropathy. The strength and novelty of this research are the prospective nature and large size of the sample and the use of multiple standardized validated comparators of nerve function (NCS, VPT, SWMF, and DNS).

Diagnosing diabetic neuropathy can be challenging as different nerve fiber populations are not evenly affected. There is an emerging consensus that small nerve fibers are earliest and most consistently affected in a length-dependent fashion (14, 23, 24). This suggests that methods based on detecting small-fiber neuropathy may be particularly promising in testing for diabetic distal symmetric polyneuropathy (7).

Stimulated skin wrinkling has been studied as a simple method of small nerve fiber function, and used to detect autonomic neuropathy in diabetics and leprosy (25, 26). It has been shown to predict abnormal IENFD in patients with sensory neuropathy (4, 5), an accepted end-point for clinical trials for early stages of diabetic neuropathy (27). Based on this knowledge, our study sought to determine the use of SSW as a cost-effective, simple diagnostic tool for distal diabetic neuropathy.

Where previous studies had used clinical examination (7), or foot ulceration (28) as clinical end-points to test various screening methods, we chose NCS criteria as our reference standard. This was to give us an unequivocal cohort of distal polyneuropathy, since mimicking conditions can result in up to 30% of patients with similar clinical characteristics (29). Clinical examination, on the other hand, is limited even in the best examiners (30). Foot ulceration as a clinical end-point is not infrequently confounded by the presence of peripheral vascular disease or musculoskeletal foot deformities. NCS remains the most objective and commonly used method of evaluating polyneuropathy in research trials (31, 32). Nerve CV has also been suggested as the best predictor of foot ulceration, when in comparison to the other tests of the sensory modalities, including perception threshold testing of vibration, temperature, and pressure (33).

Our results validate our previous findings of using the cut-off point of <3 as an abnormal SSW test when in comparison to NCS-determined DSPN (1). Using this cut-off, SSW was of at least equivalent sensitivity and specificity in determining the presence of DSPN as that of VPT testing with a hand-held Biothesiometer, which was found to have the best sensitivity and specificity in comparison to the other studied methods of testing for DSPN based on ROC-analysis. Our study also showed that SSW test is dichotomizes between normal and abnormal NCS findings and normal and abnormal DNS scores.

Nerve conduction studies, while the most sensitive and specific method for detecting diabetic polyneuropathy (12), have the problem of requiring trained technologists and/or neurologists with expensive equipment to perform and interpret the studies. Furthermore, NCS do not identify small nerve fiber damage, an important component of diabetic neuropathy.

Alternative, more commonly used, screening methods include light touch testing via monofilament, or vibratory sensation testing via a hand-held biothesiometer or tuning fork (34). These tests, however, again are restricted in their assessment of mainly large fiber function, with limited value in cases of early neuropathy, other neurological comorbidities, and in the elderly (12, 35, 36). In addition, these screening methods have been rarely compared to NCS findings, but rather to predict foot ulceration (28). Despite being more readily administered than NCS, these tests still require some training and the purchase of specialized equipment.

Clinical scoring systems, despite being advocated as a possible way to assess both large and small-fiber dysfunction, are limited in their need for patient cooperation, and can be time-consuming and tedious to administer. IENFD as a screening test for small-fiber dysfunction in diabetes cannot be used as a screening tool due to its invasiveness and need for high-level expertise in obtaining and interpreting the sample. Other available tests to evaluate small-fiber function include pain perception (Neurotip), qualitative temperature perception (Tiptherm rod), thermal perception threshold (TPT) testing, and sudomotor function tests, as well as more novel methods including NeuroQuick and Neuropad. Again, many of these tests involve expensive equipment and trained personnel, and lack rigorous validation (7). More recently, ultrasonography of the posterior tibial nerve has been studied as a screening method for DSPN (37). Other than requiring trained sonographers, and the need to purchase an ultrasound machine, sonography demonstrated a lower sensitivity than SSW testing (69 vs. 81.3%).

Eutectic mixture of local anesthetic-induced SSW testing has a theoretical advantage of being able to screen for both early and late diabetic neuropathy based on the concept of small nerve fiber dysfunction being affected early. In out study we were not able to confidently categorize patients recruited into early of late neuropathy and so were not able to test this theory. EMLA-induced SSW testing is easy to perform, inexpensive, and requires no specialized equipment except tubes of EMLA cream and sticky tape. EMLA-induced SSW is based on water-immersion skin wrinkling, and tests sympathetic nerve fibers which regulate digit pulp vasoconstriction (2). In addition, the skin response to EMLA follows a more linear response than water wrinkling, and persists for over 90 min, allowing sufficient time for grading of the wrinkling by the observer (2). As both our results presented here and earlier studies have shown, SSW demonstrates good repeatability, and can be achieved with minimal training (1).

Papanas and Ziegler proposed that an ideal test for diabetic neuropathy should fulfill the following criteria: (a) high sensitivity, specificity, and reproducibility; (b) be easy to use; (c) contribute to early diagnosis of neuropathy; and (d) be cost-effective (7). In this, SSW fulfills the criteria of being reproducible, easy to use, cost-effectiveness, and potentially the ability to detect early diabetic neuropathy. While the sensitivity of SSW is fairly high, specificity is somewhat lower suggesting that it may be best used as a screening tool for diabetic neuropathy.

Our work is limited by not correlating the use of SSW with the clinical end-point of foot ulceration and amputation. However, foot ulceration also results from the contributory factors of peripheral vascular disease that may confound skin wrinkling, while NCS is an objective marker of neuropathy. Our patient cohort also consists of mainly type two diabetic patients. This is not surprising, given our adult cohort of patients and a minority of patients having Type 1 Diabetes. A further issue decreasing the sensitivity and specificity of SSW is that it is performed over the fingers and not over the toes, the latter harboring the longer axons, and representing the maximum of diabetic length-dependent neuropathy. However skin wrinkling of the toes has yet to be translated into a repeatable useful test of small nerve fiber function and needs to overcome the considerable technical difficulties of performing this test on the toes.

Patients with more natural skin wrinkling, e.g., the elderly, may also make interpretation of the final wrinkling grade difficult. We had previously found that this can be circumvented by comparing the area of stimulated wrinkling with that of the adjacent control skin in order to counter over-scoring (5). Our study validates this as a reasonable method of grading skin wrinkling, given we found that the elderly were more likely to have abnormal wrinkling scores, correlating with the finding of age as an independent predictor of IENFD in healthy individuals (38). Further improvement of specificity may result if pre-existing wrinkling is subtracted from the final wrinkling score.

In this study, we also sought to look for other possible confounders affecting skin wrinkling, such as median neuropathy at the wrist, given that involvement of peripheral vasomotor fibers has been reported in carpal tunnel syndrome (39). More recently, in a smaller sample of 32 hands, carpal tunnel syndrome has been shown to result in reduced vasoconstriction (and hence skin wrinkling) over digits 3 and 4 (40). Our study, however, could not confirm this as a confounder.

To further enable the use of SSW in the screening for DSPN, studies should be performed on the relation of SSW to resultant amputation or foot ulcers. Furthermore, SSW in patients with impaired glucose tolerance may be useful since as a test, SSW could be particularly useful in subclinical diabetic neuropathy, where NCS does not detect abnormality.

In summary, our study suggests that SSW can be used as a diagnostic tool for diabetic neuropathy. Its advantages are low cost, simple administration, and easy reproducibility with minimal training. It may have a particular role in the cognitively impaired and other patients who are unable to tolerate NCS, or cooperate with questionnaires or monofilament and threshold testing. With comparable sensitivity to other diagnostic methods, SSW can be considered especially in the general practice setting, similar to urine dipstick testing for renal dysfunction, and retinal photography for eye damage.

## Conflict of Interest Statement

The authors declare that the research was conducted in the absence of any commercial or financial relationships that could be construed as a potential conflict of interest.
